# *Dirofilaria repens* infection in a dog imported to Norway

**DOI:** 10.1186/1751-0147-56-6

**Published:** 2014-01-21

**Authors:** Bente K Sævik, Einar Jörundsson, Teresa Stachurska-Hagen, Kristoffer Tysnes, Hege Brun-Hansen, Henriette C Wikström, Lucy J Robertson

**Affiliations:** 1 Department of Basic Sciences and Aquatic Medicine, Norwegian School of Veterinary Science, PO Box 8146, N-0033 Oslo, Norway; 2 Department of Food Safety and Infection Biology, Norwegian School of Veterinary Science, PO Box 8146, N-0033 Oslo, Norway; 3 Din Dyreklinikk AS, Skiringsalsveien 9, N-3211 Sandefjord, Norway

**Keywords:** Canine, Knott’s test, Molecular methods, Cytology, *Dirofilaria repens*, Import, Clinical signs, Zoonosis

## Abstract

*Dirofilaria repens* infection was diagnosed in a dog that had been imported to Norway from Hungary three years previously. The dog was a four-year-old castrated male mixed-breed dog and presented for examination of two masses on the right thoracic wall. Fine needle sampling from the subcutaneous nodules and subsequent cytological examination revealed a high number of microfilariae and a pyogranulomatous inflammation. At re-examination approximately 3 weeks later, both masses had apparently disappeared spontaneously, based on both inspection and palpation. However, examination of peripheral blood by a modified Knott’s test revealed a high number of unsheathed microfilariae with mean length of 360 μm and mean width of 6-7 μm, often with the classic umbrella handle appearance of *D. repens*. Polymerase chain reaction and sequencing confirmed the *D. repens* diagnosis. Subcutaneous dirofilariosis caused by *D. repens* is probably the most common cause of human zoonotic dirofilariosis in Europe, but currently is rarely encountered in northern countries such as Norway. However, travelling, import and relocation of dogs have increased, and thus the geographical range of these parasites is likely to increase from traditionally endemic southern regions. Increasing numbers of autochthonous cases of *D. repens* infections in dogs have been reported in eastern and central Europe. Although infection with *D. repens* often induces only mild signs or subclinical infections in dogs, they nevertheless represent a reservoir for zoonotic transmission and thus a public health concern, and, in addition, due to the long prepatent period and the high frequency of subclinical infections or infections with unspecific clinical signs, could easily be missed. Lack of experience and expectation of these parasites may mean that infection is underdiagnosed in veterinary clinics in northern countries. Also, predicted climate changes suggest that conditions in some countries where this infection is currently not endemic are likely to become more suitable for development in the intermediate host, and thus the establishment of the infection in new areas.

## Background

Transmission and occurrence of nematodes of the genus *Dirofilaria* are dependent on environmental factors. In particular, as development in the mosquito intermediate host is temperature dependent [1], ceasing at temperatures below 18°C and being more rapid at higher temperatures (8-10 days at 28-30°C, but 16-20 days at 22°C), infection is more common in southern Europe, and is rarely encountered in northern countries such as those of Scandinavia. However, travelling, import and relocation of dogs have increased, partly due to harmonization of European rules, and thus the geographical range of these parasites is likely to increase from traditionally endemic southern regions. Increasing numbers of autochthonous cases of *Dirofilaria repens* infection in dogs have been reported in eastern and central Europe in recent years: Czech Republic [2], Slovakia [3], Austria [4], The Netherlands [5], Germany [6-8] and Poland [9].

Nevertheless, lack of experience and expectation of these parasites may mean that infection is underdiagnosed in veterinary clinics in northern countries. This is important, not only as *Dirofilaria immitis* (heartworm) infection is a serious and potentially fatal disease in dogs and cats, but also because subcutaneous dirofilariosis, caused by *D. repens* is probably the most common cause of zoonotic dirofilariosis in Europe. Furthermore, predicted climate changes suggest that conditions in some countries where this infection is currently not endemic are likely to become more suitable for development in the intermediate host, and thus the establishment of the infection in new areas [10-13].

Although infection with *D. repens* often induces only mild signs or subclinical infections in dogs, they nevertheless represent a reservoir for zoonotic transmission and thus a public health concern, and, in addition, due to the long prepatent period and the high frequency of subclinical infections or infections with unspecific clinical signs, could easily be missed.

The purpose of the present report is to describe the clinical presentation of a case of *D. repens* in a dog imported to Norway and that had presumably lived in Norway for a considerable period with this infection, and thereby to draw attention to the possibility of *D. repens* infections in dogs imported to non-endemic areas and which may result in the establishment of endemic infection should climatic and environmental conditions be suitable.

## Case presentation

### Clinical findings at presentation

A four-year-old castrated male mixed-breed dog was presented for examination of two masses on the right thoracic wall. The dog was a former stray dog and had been imported to Norway from Hungary at the age of 1 year, approximately three years previously. Except for an intermittent lameness in the right front leg, the dog had otherwise been healthy.

On physical examination, the dog was bright, alert and responsive. The cutaneous masses extended into the subcutaneous tissue and were freely movable, firm, painless and with intact skin and coat. One mass was circular and measured approximately 4.5 cm in diameter; the other one was elongated and irregular, with length 5 cm and width ranging from 1 to 2 cm. The physical examination was otherwise unremarkable.

### Cytological findings

Material collected by fine needle sampling (with a 23 gauge needle) was smeared on slides and air-dried before fixation and staining with modified Wright (Hema-tek^®^2000, Bayer, Leverkusen, Germany) at the Central Laboratory, Norwegian School of Veterinary Science (NVH).

**Figure 1:**
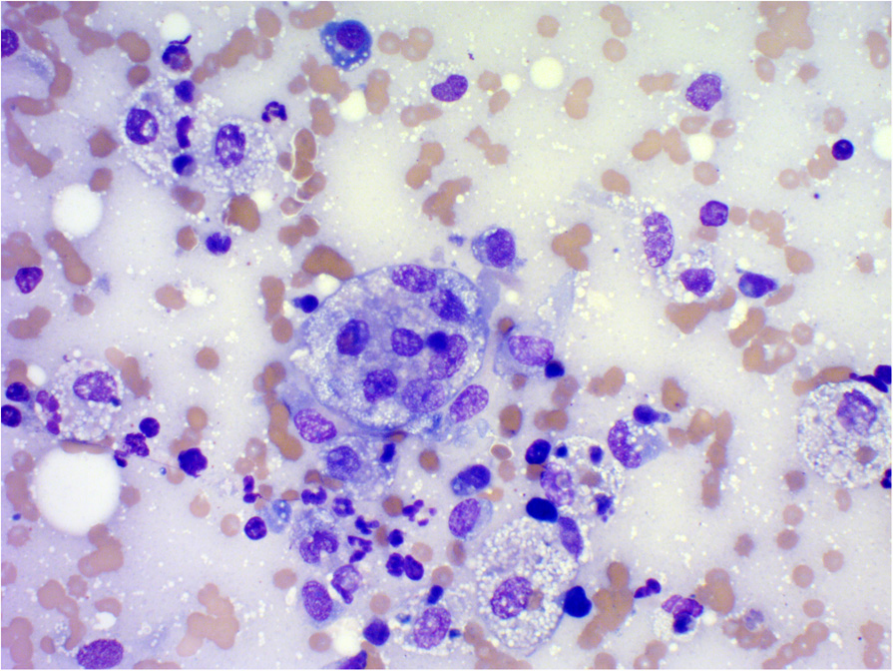
**Fine needle sample from a subcutaneous mass on the right thoracic wall.** Blood contaminated basophilic background. Many macrophages and non-degenerate neutrophils. Macrophages exhibiting cytophagia. A low number of plasma cells, an eosinophil and a multinucleated macrophage. Modified Wright’s, original magnification 400×.

**Figure 2:**
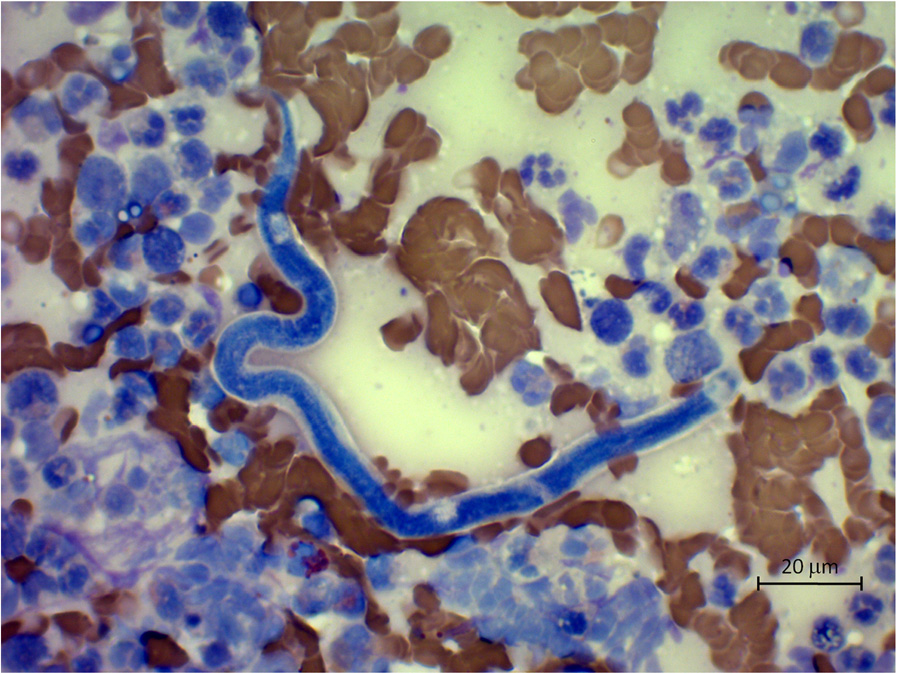
**Fine needle sample from a subcutaneous mass on the right thoracic wall.** Microfilaria with an obtuse cephalic end and a sharp and filariform tail dispersed in an inflammatory exudate. Modified Wright’s, original magnification 630×.

Ten glass slides from the two masses were examined microscopically; 5 from each mass. There was a moderate to high number of erythrocytes in a pale basophilic background. Smears were highly cellular and dominated by non-degenerate neutrophils and macrophages, often exhibiting cytophagia. A low number of plasma cells and scattered multinucleated cells, eosinophils and mast cells were also noted. In addition, numerous larvae, ranging from 0 to 11 per field (×10 objective), were identified in smears from both masses. Larvae were estimated to measure approximately 300 × 6-7 μm and exhibited an obtuse cephalic end with nuclei and a sharp and filariform tail (Figures 1 and 2).

The cytological diagnosis was a nematode/filarial worm infection with pyogranulomatous inflammation. Differential diagnoses included infections with *D. repens*, *D. immitis* (in aberrant location), *Acanthocheilonema reconditum*, *Acanthocheilonema dracunculoides*, *Onchocerca lupi* or *Cercopithifilaria* spp.

Based on cytological findings, a re-examination where the patient was examined more thoroughly, including clinical chemistry and hematological analyses, a screening for infectious diseases associated with travelling (“travel diseases”), a fecal examination for endoparasites and a Knott’s test for microfilaremia was scheduled. Additionally, it was intended that skin snip samples would be taken to investigate for dermal microfilariae [14] and excisional biopsy for histopathological examination.

### Clinical findings at re-examination

At re-examination, approximately 3 weeks later, both masses had apparently disappeared spontaneously (i.e. without any treatment); they were not possible to identify either by inspection or palpation. Consequently, skin snip samples and biopsies were not taken.

The rest of the physical examination was unremarkable. Lateral and ventrodorsal radiographic projections revealed no enlargement of internal organs or other significant radiographic changes in the thoracic and abdominal cavity. Serum, EDTA blood and freshly made blood smears were sent to the Central Laboratory (NVH) for biochemical and hematological analyses. On the “standard” biochemical panel and complete blood count (CBC), no deviations from the reference intervals were noted. Furthermore, the CRP was 1.0 mg/L (reference interval: 0-15.0 mg/L). Morphological examination of blood smears revealed a low number of microfilariae.

### “Travel disease profile”

In the “Travel disease profile” (IDEXX Vet Med Labor, Ludwigsburg, Germany) negative results were reported for *Ehrlichia canis* antibodies, *Leishmania* antibodies (ELISA), Macrofilaria (ELISA), and *Babesia* spp (real time PCR).

### Parasitological findings

A fresh fecal sample was examined at the parasitology laboratory, NVH by standard techniques for endoparasites, including McMaster egg counting technique, sucrose flotation on direct smears, and immunofluorescence antibody test for *Cryptosporidium* and *Giardia* infection. All results were negative.

**Figure 3:**
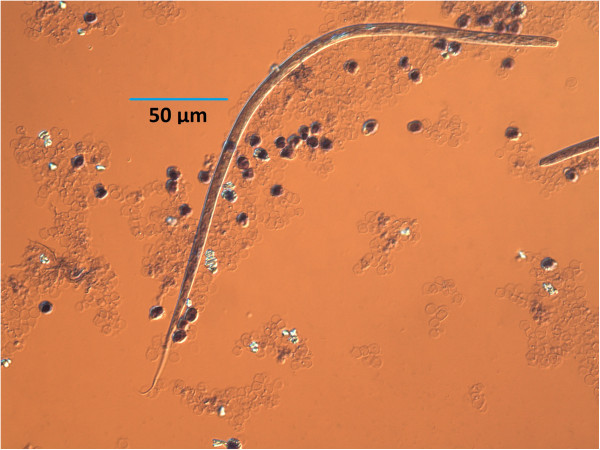
**Microfilaria in Knott’s test.** Typical example of microfilaria seen in Knott’s test as visualized under DIC. Note classic umbrella handle tail morphology.

The modified Knott’s technique was conducted by standard procedures. In brief, 1 ml of blood was mixed with 9 ml of 2% formalin and mixed well. Following centrifugation (5 min at 1500 rpm), the supernatant was decanted off and a drop of 1% aqueous methylene blue added to the sediment. Following mixing, 20 μl sub-samples were examined by microscopy and microfilariae were observed (300 per 100 μl of sediment), with mean length of 360 μm and mean width of 6-7 μm (derived from measurements of 10 randomly selected microfilariae). The microfilariae were unsheathed with obtuse cephalic ends and a sharp tail, often with the classic umbrella handle appearance of *D. repens* (Figure 3).

Due to the overlap in size of *D. repens* blood microfilariae and those of other filarial worms, a molecular test was performed to confirm the diagnosis. Sediment from the Knott’s test was freeze-thawed once, and DNA extracted using QIAamp DNA mini kit (Qiagen GmbH, Hamburg, Germany) according to the manufacturer’s instructions. Amplification of DNA at the cytochrome oxidase subunit I (*cox I*) gene was performed as described using primers first described by Casiraghi *et al.*[15] (COIintF (5-TGATTGGTGGTTTTGGTAA-3) and COIintR (5-ATAAGTACGAGTATCAATATC-3) and cycling conditions as follows: 94°C 45 sec, 52°C 45 sec, and 72°C 90 sec for 40 cycles, followed by holding at 4°C. Duplicate samples were run, with water as a negative control. Visualization of products by gel electrophoresis demonstrated products of the expected size (approximately 650 bp) for each sample, and the products were purified (High Pure PCR purification kit, Roche Diagnostics GmbH, Mannheim, Germany) and sequenced on both strands at a commercial laboratory. Electropherograms were clean, and BLAST comparisons in GenBank showed that the sequences had 100% identity with previously submitted partial sequences of the *D. repens cox I* gene (e.g. GenBank Accession number JF461458), thereby confirming the *D. repens* diagnosis.

### Treatment and follow-up

After the diagnosis was confirmed, the local Norwegian Food Safety Authority was notified. They instructed the owner to treat the dog with monthly application of moxidectin 2.5%/imidacloprid 10% (Advocate^®^ spot-on, Bayer Animal Health GmbH, Leverkusen, Germany) in an attempt to eliminate microfilaremia. The dog was subsequently lost for follow-up.

## Discussion

To the best of our knowledge, only one case of *D. repens* infection in a dog living in Scandinavia has previously been published in the scientific literature. Bredal *et al.*[16] described a subcutaneous granuloma on the chest of a dog imported from South Africa to Norway in which an adult *D. repens* was identified. In this case, blood microfilariae were not identified despite repeated efforts, reportedly due to infection with a single female worm with unfertilized eggs. The number of dogs travelling, relocated or imported to Norway and the rest of Scandinavia appears to have been increasing in recent years. Specifically, a number of canine protection organizations have been facilitating the import of stray dogs to Norway, in particular from Hungary and Romania [17], where *D. repens* is endemic.

In a survey by Pantchev *et al.*[18], *D. repens* was the most common canine filarial infection imported into Germany between 2008 and 2010, and originated from eleven European countries, most commonly Hungary, but also Greece, Italy, Spain and Romania. Generally, filarial screenings within “travel disease profiles” investigate solely for heartworm (*D. immitis*) using an antigen detection test that does not detect *D. repens*. Pantchev *et al.*[18] commented that the awareness of German veterinary surgeons regarding other filarial infections is low, so infections with filarial species other than *D. immitis* are probably underdiagnosed. The same is probably true in most other northern European countries, including Norway, and consequently *D. repens* infections in dogs imported to Norway is probably underdiagnosed.

Recently, Albanese *et al.*[19] described the clinical and histopathological features of *D. repen*s infections in 16 dogs in Italy presenting with cutaneous nodules. In each case one to six nodules, measuring from 0.5 to 4 cm and located in different anatomical sites, were reported. No other clinical signs were noted. Adult worms were confirmed in the nodules in 15 out of 16 dogs, and microfilariae were observed in 13 out of 13 of the animals for which results of fine needle aspirates were available. In other studies, several, albeit nonspecific, cutaneous findings have been described in canine cases of *D. repens* infection [20-23]. However, often the presence of *D. repens* in the skin lesions was not confirmed and the correlation between clinical signs and occurrence of worms is circumstantial or based on regression of clinical signs after treatment. Furthermore, findings in one study suggest that only 12% of dogs with *D. repens* infection presented with cutaneous nodules [22]. Adult *D. repens* worms may reside for up to 4 years in subcutaneous tissue. In our Norwegian case, the dog had presumably been infected in Hungary prior to import to Norway, 3 years prior to diagnosis, and upon questioning, the owners remembered a small lump in the skin on the left side of the neck some months previously that had disappeared spontaneously. Other lesions may have been undetected because of small size and/or deep subcutaneous location. Migrating subcutaneous nodules in *D. repens* infection have been reported from humans who are aberrant hosts [24], as the worms move through the subcutaneous tissue. Presently the type of immune response dogs mount against *D. repens* is unresolved and the components of the worm and/or larvae that are responsible for inducing subcutaneous inflammation have not been identified. Although our case was not examined for evidence of *Wolbachia* infection, bacteria from the genus *Wolbachia* have an endosymbiotic relationship with *Dirofilaria* spp. that could affect the inflammatory features and thus the clinical outcome of infection [25]. Finally, one might speculate whether fine needle sampling may have provoked regression of the nodules in our case, although considered unlikely.

Knott’s test is recommended for the detection of *D. repens* microfilariae. However, in the study by Albanese *et al.*[19] only 4 out of 12 dogs tested by Knott’s test were positive. Thus, use of a variety of tests, including Knott’s test for the detection of blood microfilariae and fine needle sampling of subcutaneous nodules with subsequent cytological examination, might be considered as the most appropriate diagnostic approach. As there are overlaps in size between filarial species, molecular methods are useful for confirming or refuting diagnostic inferences from morphological information.

Diagnosis of subcutaneous dirofilariosis in the dog can be problematic and in-clinic tests, such as those for *D. immitis*, do not currently exist. These diagnostic challenges, along with long incubation period, mild and transient clinical signs, and, in northern countries, lack of diagnostic experience and expectation of this infection, are likely to result in lack of diagnosis of many infections. This enables the infection to spread in epidemic areas and to be introduced into new areas. In recent years, increasing numbers of autochthonous cases of *D. repens* infection in dogs have been reported in Europe [2-9]. For establishment of the *D. repens* infection cycle, the presence of mosquitoes, primarily from the genera *Aedes*, *Anopheles* and *Culex*, as well as suitable climatic conditions (sufficiently high temperature for a sufficient period for the development of infective larvae within the mosquito) are necessary. While these mosquito genera are widespread in most countries in Europe, included in northern countries such as Norway, appropriate climatic conditions occur less frequently. However, with the current predicted climate change scenarios, this is likely to change, and therefore veterinarians should be on the alert to ensure that the infection is not introduced, otherwise it is increasingly likely to become established.

Treatment of dogs diagnosed with *D. repens* infection is desirable to eliminate the microfilariae and reduce further spread of infection and establishment of the infection in new regions. Up until now, sparse information has been available on treatment and control of *D. repens* infection. In a recent field study the monthly application of moxidectin 2.5%/imidacloprid 10% (Advocate^®^) spot-on reportedly eliminated microfilariae for up to 6 months, following 6 months of treatment [26]. Hellman *et al.*[27] and Genchi *et al.*[28] also reported that the application of moxidectin 2.5%/imidacloprid 10% was effective in the treatment and prevention of canine *D. repens* infection. Moreover, the use of doxycycline and ivermectin in combination has been suggested as a good option for eliminating *D. repens* microfilariae from dogs [7,29]. Unfortunately the dog in our case was lost to follow up.

## Conclusion

Veterinarians in countries where *D. repens* is not considered endemic should be alerted to the potential for the import of this infection with dogs, and nodular skin lesions from such dogs should be carefully evaluated. As the dog in the case described here had presumably been living in Norway for around 3 years with the potential to transmit the infection further, and probably other imported dogs or dogs that have visited endemic areas continue to live in Norway with the infection un-diagnosed, the possibility that transmission has occurred and thus of this infection occurring in dogs that have not visited endemic areas should not be excluded. That is, *D. repens* infection should be considered a differential diagnosis in all dogs presenting with subcutaneous nodular lesions regardless of travel history. In terms of diagnosis, cytological evaluation seems to be a sensitive diagnostic method for detection of microfilariae in nodules in the subcutaneous tissue, and should be supported by other diagnostic tests, including Knott’s test. Where possible, and particularly where experience with microfilaria morphology is limited, the diagnosis can be readily confirmed with molecular testing.

## Abbreviations

bp: Basepair; CBC: Complete blood count; CoxI: Cyclooxygenase 1; CRP: C-reactive protein; DIC: Differential interference contrast; EDTA: Ethylene diamine tetraacetic acid; ELISA: Enzyme-linked immunosorbent assay; NVH: Norwegian School of Veterinary Science; PCR: Polymerase chain reaction.

## Competing interests

The authors declare that they have no competing interests.

## Authors’ contributions

BKS and LJR contributed to the work up of the case. BKS drafted the manuscript, with contribution and input from LJR. EJ, HBH and BKS performed the cytological examination and interpretation. TSH, KT and LJR performed the parasitological examinations, including PCR and sequence analysis. HCW is the referring clinician of the case. All authors read and approved the final manuscript.
